# Insights from a Patient-Centered Lung Cancer Navigation Program in a Low-Resource Community

**DOI:** 10.3390/curroncol32090491

**Published:** 2025-09-01

**Authors:** Tanyanika Phillips, Anjaney Kothari, Africa Robison, Jeffrey Mark Erfe, Dan J. Raz

**Affiliations:** 1 Department of Medical Oncology and Developmental Therapeutics, City of Hope National Medical Center, Duarte, CA 91010, USA; 2 Division of Thoracic Surgery, Department of Surgery, City of Hope National Medical Center, Duarte, CA 91010, USA; akothari@coh.org (A.K.); jmerfe@gmail.com (J.M.E.); draz@coh.org (D.J.R.); 3 Sheri and Les Biller Patient and Family Resource Center, Department of Supportive Care Medicine, City of Hope National Medical Center, Duarte, CA 91010, USA; arobison@coh.org

**Keywords:** telehealth lay navigation, lung cancer, low-resource community, patient-centered, practical barriers

## Abstract

Barriers to cancer care, including transportation and Internet insecurity, are of special concern in low-resource communities. A patient-centered, telehealth-based, barrier-focused lay navigator program may mitigate such barriers. We share insights from a quality improvement project wherein we developed and delivered a lay navigator program in a low-resource community in the Mojave Desert. We identified 68 patients scheduled for lung cancer detection/management at our institution, 55 of whom completed a barrier assessment, enrolled in the program, and could be evaluated. Participants were predominantly older (76%), White (84%), had a cancer diagnosis at enrollment (69%), and lived in socioeconomically disadvantaged neighborhoods. Thirty-three (60%) patients had ≥1 barrier, the most common being transportation (31%), Internet (24%), and financial (24%) concerns. These barriers were more frequent among patients with a lung cancer diagnosis at enrollment. Crisis-focused and after-hours encounters were more frequently initiated by older and advanced cancer patients. Transportation and Internet concerns were significantly associated with missed appointment rates. While the scope of our findings is limited, the delivery of a telehealth-based, barrier-focused lay lung navigator program in this low-resource setting was feasible. Neighborhood context and barrier resource planning are important for the implementation of similar programs within our institution’s clinical practice network.

## Simple Summary

Low-resource communities often face barriers to cancer care that may be resolved through patient navigation led by a lay navigator equipped with barrier-specific resources. In this manuscript, we share insights from our experience developing and delivering a lung cancer-focused lay navigator program in a low-resource community in the Mojave Desert. We identified transportation concerns, Internet needs, and financial concerns as the most common barriers to care among patients scheduled for lung cancer detection/management at our institution. Patients who had a lung cancer diagnosis at enrollment were more likely to report barriers to care. Time spent navigating a patient was longer for patients with a greater number of barriers. Crisis-focused and after-hours navigation calls were more frequently initiated by older and advanced cancer patients. Overall, we identified neighborhood context and barrier resource planning as important considerations to improve the implementation of our patient navigation program within our institution’s clinical practice network.

## 1. Introduction

Low-resource communities receive disproportionately worse cancer treatment and delivery of services than high-resource communities, primarily due to inequitable access to the benefits of scientific advances [1,2,3]. For the past three decades, this fact, enshrined in Freeman et al.’s Discovery–Delivery Disconnect principle, has guided community-based patient navigation programs seeking to promote timely access to care while eliminating barriers to care [4]. However, as patient navigation programs have expanded, so has the diversity of barriers to healthcare. For example, with the onset of the COVID-19 pandemic in 2020, the ‘Digital Divide’, a term referring to limited access to digital information and technology in low-resource communities, emerged as a significant new barrier [5]. Traditional barriers (such as lack of access to transportation) and new barriers (such as lack of Internet access or digital literacy) both have multidimensional effects on patient outcomes. For example, in southern California, geographic barriers to comprehensive care, and decreased access to or use of telehealth, are associated with an increased reliance on local emergency room care, missed appointments, and delays in care in low-income populations [6,7,8,9].

Patients with lung cancer often face significant barriers to care due to disparities in access to screening and the shame and stigma associated with smoking [10,11]. A growing body of research on patient navigation in lung cancer care has identified additional barriers, including communication issues, financial concerns, challenges accessing telehealth, transportation difficulties, and mental health concerns, among others [12,13,14]. In addition to these practical and psychosocial barriers, an undersupply of multidisciplinary professionals needed for complex cancer management further hinders access to quality cancer care in low-resource communities [15].

Using patient navigation to alleviate practical barriers, such as transportation or Internet challenges, and coordinating complex cancer care may improve the quality and timeliness of healthcare for patients with lung cancer in low-resource communities. The success of patient navigation is rooted in patient-centeredness, defined by the National Academy of Medicine (USA) as “providing care that is respectful of, and responsive to, individual patient preferences, needs, and values, and ensuring that patient values guide all clinical decisions” [16,17]. Lay navigators have the potential to improve patient-centeredness of healthcare, because they are trained personnel with a strong cultural connection and attunement to the community, a familiarity with the patient geography, and the ability to respond to patients on a more personal level, empower them, coordinate and navigate access to care, and assist with alleviating practical barriers in care [18,19,20,21]. The literature on lay navigation programs for patients with lung cancer is sparse, with existing programs having focused either on (1) improving lung cancer screening by resolving barriers to screening, without providing resources to overcome psychosocial and practical barriers to post-diagnosis care [22]; or (2) providing resources and referrals and assessing patient satisfaction without evaluating care outcomes, such as adherence to care or time to diagnosis/treatment [23]. Thus, there is a need to develop barrier-focused lay navigator programs for patients in need of screening or care for lung cancer and evaluate their impact on patient and care outcomes.

Importantly, prior lay navigation programs have primarily involved telephonic or mixed interventions. This is pertinent, given that virtual navigation can increase patient access to cancer navigation and education [24]. Moreover, the use of telehealth approaches among patients with lung cancer has been shown to be associated with significantly higher quality of life, including lower anxiety and depression, than usual care [25]. Notably, telehealth, as defined by the US Department of Health and Human Services, covers the use of diverse telecommunications technologies, including landline and wireless communications, Internet, videoconferencing, and streaming media, to facilitate long-distance healthcare services [26].

Guided by the findings and principles discussed above, we initiated the City of Hope (COH)-Antelope Valley (AV) Lung Cancer Navigator Program, a telehealth-based, barrier-focused lay navigator program for patients with newly diagnosed lung cancer or imaging findings concerning lung cancer in AV, a low-resource community located in the western region of the Mojave Desert. We undertook this navigator program as a quality improvement (QI) project to gain insights into (1) thoracic cancer care barriers perceived by patients at COH; (2) healthcare resource challenges and neighborhood disadvantages related to vulnerable communities served by COH [27]; and (3) the demographic and clinical characteristics of patients who need patient navigation the most. Herein, we summarize the insights gained from the delivery of this telehealth-based, barrier-focused lay navigator program.

## 2. Methods

The COH-AV Lung Cancer Navigator Program was planned, delivered, and evaluated over 34 months from February 2022 to December 2024. Eligible patients required a presentation of suspicious radiographic findings in the lung(s) or a diagnosis of lung cancer under evaluation for treatment at COH-AV at the time of enrollment. This QI project was reviewed and approved by the COH Institutional Review Board (IRB number: 21654, approval date: 19 November 2021). The QI project was carried out in full compliance with ethical standards and institutional policies. Reporting adhered to the Standards for Quality Improvement Reporting Excellence (SQUIRE) 2.0 guidelines [28]. For publication, only de-identified, aggregate data are presented, with suppression or aggregation of small cell counts (≤5) and the removal of direct identifiers.

### 2.1. Setting

The QI project was delivered at COH-AV, a clinical practice site of medical oncologists, radiation oncologists, and non-thoracic surgical specialists. COH-AV, a part of the COH medical enterprise, is located in Lancaster, California, within AV, a 3000-square-mile region at the western tip of the Mojave Desert that straddles the northern Los Angeles and southern Kern counties. The area is a low-resource community, with fragmented health care delivery, impaired neighborhood infrastructure, high tobacco use [29], and suboptimal or limited social resources [30,31]. AV has the highest lung cancer death rate in Los Angeles County [29]. The region is predominated by socioeconomically disadvantaged neighborhoods, with 40% of its population living below 200% of the federal poverty line [32,33]. While the central catchment area of AV is approximately 500,000 residents, COH-AV serves both urban and rural regions across six California counties (Los Angeles, San Bernardino, Kern, Inyokern, Inyo, and Mono counties). More than a third of COH-AV patients travel more than 50 miles (60 min) one way, and more than a third of those patients drive more than 190 miles (at least 3 h) one way to receive cancer care. Patients travelling for surgical thoracic care at COH-Duarte, the hub of the COH enterprise where thoracic surgical services are available, travel a minimum of 80 miles from COH-AV for care.

### 2.2. Intervention

The intervention included telephone-based encounters with the lay navigator to assess barriers, listen to concerns, and provide coordination of care. The program navigator was an individual who lived in the AV community for more than 30 years and worked within the COH health care system for 11 years as a medical assistant and administrator. The navigator received (1) 160 training hours on the skills, role, responsibilities, and scope of a lay patient navigator; (2) 30 h of Collaborative Institutional Training Initiative (CITI) training on research human subjects, ethics, compliance and regulatory management, privacy, and Health Insurance Portability and Accountability Act (HIPAA) regulations [34]; as well as (3) education on cultural competence, similar to other lay navigators, intensity of training regarding hours [35]. Additionally, the navigator had formal training as a life coach, equipping them with skills in empathy and problem solving, time management, critical thinking, multitasking, collaboration, and communication, consistent with the essential navigator skills discussed in the published literature [19,36].

The lay navigator identified patients either by screening the schedules of COH-AV medical oncology providers or from direct provider referral to the navigator program via email, phone, or medical record (Epic^®^, Epic Systems Corporation, Verona, WI, USA) communication [37]. English-speaking patients anticipating diagnostic or treatment intervention were eligible. Navigation included an initial telephone session, during which amenable patients were enrolled into the program and completed a barrier assessment (Table S1). While the barrier assessment utilized by the navigator was not validated, it was developed to gather preliminary insights on barrier challenges at COH in collaboration with our patient partners, who reviewed the initial draft. The navigator also conducted a mock interview with one of the patient partners for feedback. If barriers were identified, follow-up encounters were scheduled by telephone or in person to provide resources, services, coordinate care (assist with appointments or communication with oncology care team), or offer referrals to tobacco cessation counselors, social workers, or financial advisors. Data on actual patient attendance/follow-up of referrals could not be obtained. For each patient, the program concluded at the end of patient management (diagnostic intervention if patients presented with suspicious radiographic findings; cancer therapy if patients had a lung cancer diagnosis) for up to six months following enrollment. The navigator also participated in activities to promote provider awareness about the program and to enhance patient recruitment and enrollment. In addition, navigator brochures were distributed to community healthcare providers, which included information about the Navigator Program and contact information. Activities included outreach events to primary care providers and community members. Navigator encounter data were entered into the program, REDCap database (Vanderbilt University, Nashville, TN, USA), an electronic data capture tool hosted at COH [38,39].

**Figure 1:**
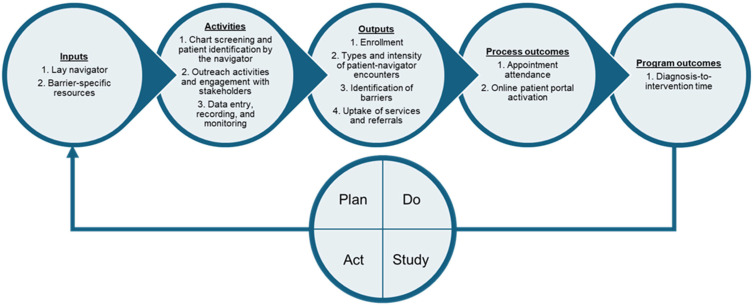
Logic model of inputs, activities, outputs, process outcomes, and program outcomes to guide the PDSA cycles implemented in the QI project.

In addition to the community site lung navigator, the core QI team included the community site lung navigator program physician champion and a collaborating thoracic surgeon. Additionally, two patient partners, who were lung cancer survivors living in AV, provided input on program development and delivery. Participants included patients, COH-AV treating physicians, medical staff assistants, a social worker, financial counselors, tobacco specialists, and the QI team. The Rapid Cycle Research framework was used to inform the QI project delivery. This framework consists of six phases: (1) preparation; (2) problem exploration; (3) knowledge exploration; (4) solution development; (5) solution testing; and (6) implementation and dissemination [40]. We used Plan-Do-Study-Act (PDSA) [41] cycles for phases 3–5 of the framework over the 34-month project timeline. The PDSA cycles were guided by a logic model of inputs, activities, outputs, process outcomes, and program outcomes, outlined in Figure 1. PDSA cycles included regular virtual meetings with the QI team, staff, and patient partners on navigator competency and skill evaluation, patient experience reflections for program adaptation, process and standard operating procedures, intensity of the program, patient stakeholder involvement in program awareness, staff participant huddles, and program activities, challenges, and triumphs.

The present manuscript is limited to describing inputs, activities, program outputs, and process outcomes. Program outcomes, including program impact on timeliness of care (time to obtaining biopsy or treatment) and patient experience, will be shared in another publication.

### 2.3. Analysis

Principal inputs included gift cards and Wi-Fi-enabled iPad^®^s to help with transportation or lack of access to telehealth or the Internet. While the navigator’s encounters with the enrolled patients were primarily completed telephonically, patients with an Internet access barrier were offered Wi-Fi-enabled iPad^®^s to help them access their patient portals, schedule appointments, view test results, communicate with the provider/care team, etc. Quality indicators were measured to describe navigator activities, program outputs, and process outcomes. Program outputs (Table S2) included the number of patients identified for and enrolled in the program, number and intensity of navigation encounters, number of patients with barriers, number of patient barriers identified, and number of patient services and referrals rendered. Process outcomes (Table S2) observed included number of appointments attended (or missed), and number of patients who activated their online patient portals, in accordance with national metrics and standards [42,43]. Descriptive statistics, such as means and medians, were used for continuous variables, and frequencies and percentages were used for categorical variables to describe patient and disease characteristics. Program enrollment, program activities, and patterns of program changes and adaptations were tracked using a run chart. Patient charts were audited to estimate patient engagement as a process outcome (frequency of appointment attendance and online patient portal activation) during concomitant navigator program delivery (Figure 1).

Healthcare resources and neighborhood disadvantages were mapped using ArcGIS Pro (Esri Inc., Redlands, CA, USA). Travel distances between ZIP Code centroids of patient residence and COH-AV/COH-Duarte were calculated using ArcGIS StreetMap Premium North America, version 2024.4 (Esri Inc.). The Area Deprivation Index (ADI), a metric that ranks neighborhood socioeconomic disadvantage using measures of income, education, employment, and housing quality [44], was used to match patient residential ZIP Code areas with neighborhood disadvantage. Specifically, state-level percentiles for ADIs (2022) at the ZIP Code Tabulation Area were obtained from the Neighborhood Atlas^®^ (Center for Health Disparities Research, University of Wisconsin School of Medicine and Public Health). Data on the number of thoracic surgeons by ZIP Code were obtained from the National Provider Index files curated by the Centers for Medicare and Medicaid Services (USA).

The Fisher’s exact test was used to evaluate statistical associations between (1) barrier frequency and sociodemographic and clinical variables; (2) the number of missed appointments and sociodemographic characteristics, clinical characteristics, and types of barriers; and (3) mode of patient recruitment and demographic variables. One-sided *p*-values were calculated for 2 × 2 contingency tables. Non-parametric Spearman correlation analysis was conducted between barrier frequency and navigation intensity. *p*-values ≤ 0.05 were considered statistically significant. All analyses were performed using GraphPad Prism (v. 10.4.1).

## 3. Results

### 3.1. Patient Enrollment

**Figure 2:**
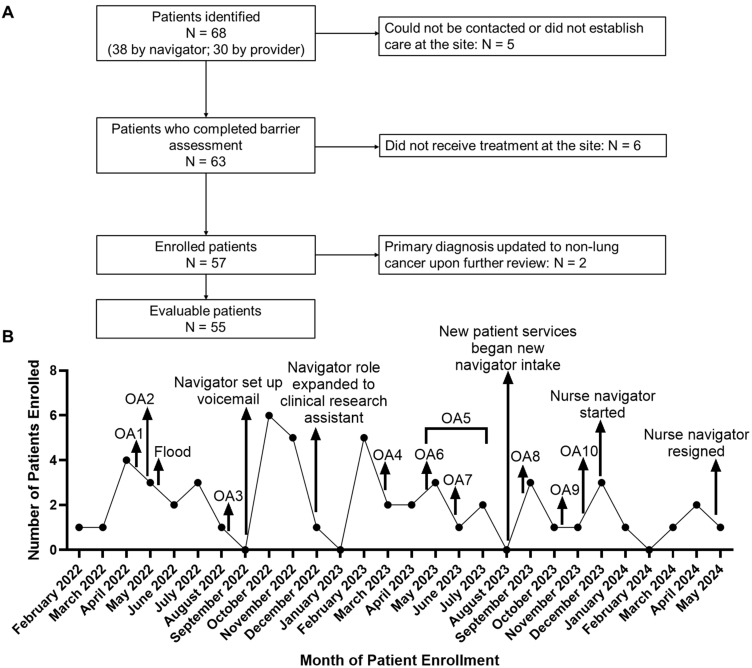
(**A**) Patient enrollment schema. (**B**) Program patterns and adaptations, depicted by the number of patients enrolled in the navigator program by month. Arrows indicate the points of occurrence of important activities or events during the navigator program. Primary care outreach activities (OAs): OA1—Annual Lancaster Poppy Festival; OA2—External Community Physician Outreach; OA3—Community Outreach Lung Cancer Awareness Dinner; OA4—Health equity in action; OA5—brochures introduced and distributed by physician liaison/marketing team; OA6—Senior and Aging Adults Resource Fair; OA7—Lung Cancer Awareness Presentation at Independent Senior Living Facility; OA8—Community Outreach at local church; OA9—Virtual presentation on Lung Cancer Screening Program to Mammoth providers; OA10—COH Marketing Team distributed emails to be sent to potential referring physicians to promote program.

Figure 2A depicts a flow diagram of patient identification, barrier assessment intake, enrollment, and evaluability. A total of 68 patients were identified for the program by the navigator (N = 38) or referred to by providers (N = 30). Patient demographic characteristics, including age, gender, race, and ethnicity, were not significantly associated with the mode of recruitment (provider referral vs. navigator screening; *p* > 0.05; Table S3). Sixty-three patients completed initial encounters for barrier assessment, and 57 patients were enrolled in the program between February 2022 and May 2024. The primary diagnoses of two of the enrolled patients were reconfirmed as non-lung cancer, resulting in a total of 55 evaluable patients (Figure 2A). Thirty-eight of these patients had lung cancer at enrollment, 16 (42.1%) of whom had a stage I or II diagnosis (Table 1). The remaining 17 patients had suspicious radiographic findings (i.e., lung mass or nodule) but did not have a lung cancer diagnosis at the time of enrollment.

Various steps were taken throughout the program to boost patient enrollment, including participating in ten outreach activities. Figure 2B depicts trends in patient enrollment over the course of the navigator program. The timelines of patient enrollment and important activities and events that occurred during the program were overlaid to assess the possible impact of these activities/events on patient enrollment. The occurrence of some events coincided with improved enrollment. For example, the addition of a nurse navigator was associated with increased enrollment in the initial period (December 2023). However, additional separate job duties performed by the lung navigator may have negatively impacted enrollment numbers. A direct correlation could not be established between patient enrollment and outreach activities, which were primarily designed to increase referrals to the navigation program from outside COH (primary care providers, specialists, self-referrals, etc.).

Overall, 29 (52.7%) of the evaluable patients came from moderately-to-highly socioeconomically disadvantaged neighborhoods (mean state-level ADIs > 5), 10 (34.5%) of whom resided in highly disadvantaged neighborhoods (mean state-level ADIs > 7). Moreover, the study participants primarily came from regions where access to thoracic surgical care was low. Specifically, fewer than 10 surgeons were listed as having thoracic surgery privileges at two community hospitals in AV, as verified by the medical executive staff office. These surgeons performed only 11 lung surgeries between 2022and 2024 combined for both hospitals, compared to over 600 lung resection surgeries performed at COH-Duarte between 2022 and 2024.

### 3.2. Patient Characteristics

Table 1 summarizes patient and disease characteristics for all evaluable patients. Most of the evaluable patients were female (31; 56.4%), white (46; 83.6%), not Hispanic or Latino (50; 90.9%), and 65 years of age or older (42; 76.4%). Participant ages were >30 years and <90 years, with a median of 71 years. Forty-seven (85.4%) participants had a history of smoking, fifteen of whom were active smokers at the time of enrollment.

Most of the evaluable patients (41 of 55; 74.5%) resided within 50 miles of COH-AV (Table 1). The longest travel distance between a patient’s home and COH-AV was >200 miles. However, ≥50 (≥90.9%) of the evaluable patients were located more than 50 miles away from COH-Duarte, with the longest travel distance between a patient’s home and COH-Duarte being >300 miles.

### 3.3. Types of Patient–Navigator Encounters

Each evaluable patient had at least two encounters with the navigator—an initial barrier assessment at the time of enrollment into the navigation program, and a program completion encounter. Any follow-up encounters with the navigator were classified into two categories: navigator-directed encounters and patient-/caregiver-directed encounters. Navigator-directed encounters were focused on either coordinating care or providing a barrier solution. Similarly, patient-/caregiver-directed encounters were primarily focused on seeking either a barrier solution (barrier-focused encounters) or crisis management (crisis-focused encounters; defined as symptom- or emotional need-driven encounters).

**Figure 3:**
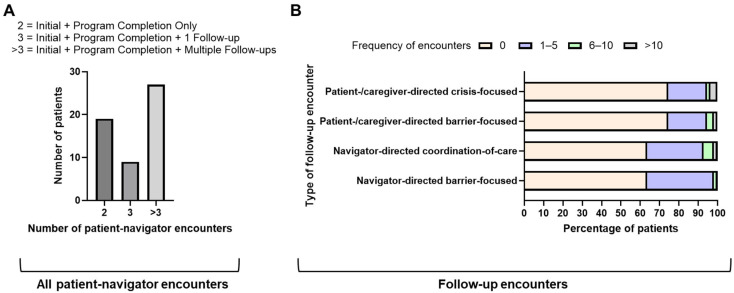
Types of patient–navigator encounters: (**A**) total patient–navigator encounters; (**B**) navigator-directed barrier-focused encounters, navigator-directed coordination-of-care encounters, patient-/caregiver-directed barrier-focused encounters, and patient-/caregiver-directed crisis-focused encounters.

Nineteen (34.5%) patients only had two mandatory encounters with the navigator (initial barrier assessment encounter and the program completion encounter) (Figure 3A). Nine (16.4%) patients had one additional follow-up encounter. Most of the patients (27/55; 49.1%) had multiple follow-up encounters with the navigator beyond the two mandatory encounters. For 20 (36.4%) patients, the navigator completed encounters that focused on providing barrier solutions (Figure 3B). Similarly, for 20 (36.4%) patients, the navigator completed encounters focused on coordination of care, with the number of such encounters exceeding five for four patients (Figure 3B).

The navigator also completed encounters initiated by a patient (or their caregiver) to seek a barrier solution or crisis management (14 patients for each type; >5 encounters of each type for 3 patients) (Figure 3B). Overall, patients with a lung cancer diagnosis in our program were more likely than those with suspicious radiographic findings to initiate calls to the navigator, whether related to barrier solutions (32% vs. 12% patients, respectively) or crisis management (29% vs. 18%, respectively). Notably, 28% (4/14) of the patients who initiated crisis-focused encounters had stage IV disease, and 100% (3/3) of the patients who initiated after-hours encounters with the navigator were ≥65 years of age with stage IV disease. Overall, the frequency of patient–navigator encounters ranged from 2 to 46, with the mean number of encounters per patient being 7 (standard deviation 8).

### 3.4. Types and Frequency of Barriers to Care

Each evaluable patient completed an initial barrier assessment either independently with the navigator or with support from caregivers. A total of 15 of the 55 evaluable patients required family/caregiver assistance to complete the barrier assessment, while the remaining 40 completed the assessment independently with the navigator. Table 2 lists the 11 barriers for which the patients were screened by the navigator. The barriers identified were either psychosocial, financial, behavioral (smoking-related), Internet access-related, or transportation-related (Table 2). Of 605 possible barriers that could have been captured (11 barriers multiplied by 55 evaluable patients), 75 barriers were captured in our study population, resulting in a 12.4% prevalence. The number of barriers was significantly associated with patients’ travel distance to COH-Duarte, with patients residing more than 50 miles away from the facility being more likely to have no or fewer (1–3) barriers (*p* = 0.006; Table S4). However, this association should be interpreted in the light of the limited number of patients (≤5) who resided more than 50 miles away from COH-Duarte in our study. Interestingly, the number of barriers in our patient population was not significantly associated with demographic (gender, age, race, ethnicity) and clinical (diagnosis, lung cancer stage) variables (*p* > 0.05).

Thirty-three (60.0%) of the fifty-five evaluable patients had at least one barrier. Transportation concerns constituted the most frequently observed barrier, affecting 31% (N = 17) of all evaluable patients. The second most frequently noted barrier was current smoking habits (15/55 patients; 27.3%), followed by Internet accessibility concerns (13/55 patients; 23.6%) and financial concerns about paying for medical care (13/55 patients; 23.6%). Notably, concerns related to transportation, Internet accessibility, and financial security were more frequent among evaluable patients who were diagnosed with lung cancer at enrollment (39.5%, 29%, and 29% of 38 patients, respectively) than those who had suspicious radiographic findings (11.8% of 17 patients for all three). Interestingly, Internet accessibility concerns (affirmative response to the question “would you be interested in borrowing an Internet-enabled iPad^®^ for telehealth appointments?”) were noted in 13 patients, even though ≤5 patients had neither a smartphone nor a computer, and only 6 patients did not have access to a reliable Internet connection at home.

The most frequently noted psychosocial barrier was the need to talk to a mental health professional (9/55 patients; 16.4%). Importantly, all patients for whom this psychosocial barrier was identified had a lung cancer diagnosis. The other psychosocial barriers identified by the navigator were concerns about paying rent or mortgage, concerns about the living situation, burden of childcare responsibilities, and being the primary caretaker of a disabled or older adult.

### 3.5. Referrals and Resources Offered

Referrals for services were offered to patients with financial, psychosocial, or tobacco cessation barriers. Overall, 31 (56.4%) of all evaluable patients were offered at least one referral to overcome a barrier (Table 2). Referrals that were availed by the patients could not be quantified since data on completed referrals could not be consistently collected. However, the navigator attempted follow-up communications encouraging referral completion, and some patients offered their reflections and experiences.

Resources offered to patients with Internet accessibility and transportation barriers included loaner Internet-enabled iPad^®^s and transportation gift cards, respectively. Internet-enabled iPad^®^s were offered to facilitate appointment scheduling, patient–provider communication, and telehealth visits. Five (38.5%) of the thirteen patients who were interested in borrowing an iPad^®^ for telehealth appointments (deemed as having Internet accessibility concerns) received iPad^®^s (Table 2). The remaining eight patients did not receive iPad^®^s for various reasons, including infeasibility of the intervention, change in care plan, or declination of service by the patient.

Transportation gift cards included Uber^®^ (Uber Technologies, Inc., San Francisco, CA, USA) gift cards or Visa^®^ (Visa Inc., San Francisco, CA, USA) gift cards. Twelve (70.6%) of seventeen patients with transportation concerns received gift cards (Table 2). The remaining five did not receive this service either because the patient or their family declined the service, the care plan changed, or the intervention was not feasible.

### 3.6. Intensity of Patient–Navigator Encounters and Their Association with the Number of Barriers Identified

Intensity of navigation is defined as the time needed to provide navigation [45]. Duration of encounters was recorded for 22 patients. For the remaining 33 patients, durations of encounters were unavailable due to one of the following reasons: (1) recording of encounter duration was initiated in October 2022 as a result of PDSA adaptive cycles to address navigator efficiency and intensity of interventions, by which time 17 of the 55 evaluable patients had already been enrolled; or (2) navigator was unable to accurately time all encounters with the patient due to the impromptu or unscheduled nature of some encounters in the clinic. Duration of encounters did not include the time spent by the navigator in following up and completing documentation and referrals after a session.

**Figure 4:**
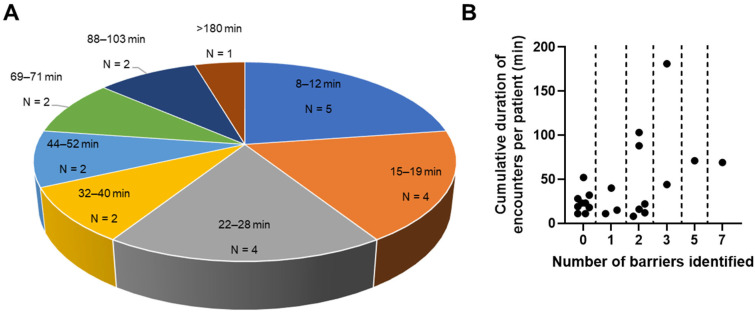
Cumulative duration of patient–navigator encounters: (**A**) frequency of patients by duration of encounters; (**B**) cumulative duration of encounters depicted against number of barriers identified.

Among the evaluable patients for whom encounter duration data were available, the median intensity of navigation was 23 min (interquartile range 35 min). Five patients had cumulative navigator encounter durations under 15 min (Figure 4A). Meanwhile, five patients had cumulative navigator encounter durations that exceeded an hour (69–181 min) (Figure 4A). Four (80.0%) of these patients had a lung cancer diagnosis at enrollment, three of whom had advanced cancer (stage III/IV). All five patients with cumulative encounter durations exceeding an hour had 2–7 barriers (Figure 4B), with transportation and Internet accessibility concerns being among the most common barriers (4/5 patients; 80%). Expectedly, a larger number of identified barriers was significantly associated with a longer cumulative duration of patient–navigator encounters (Spearman’s r = 0.39, *p* = 0.035).

### 3.7. Patient Engagement

**Figure 5:**
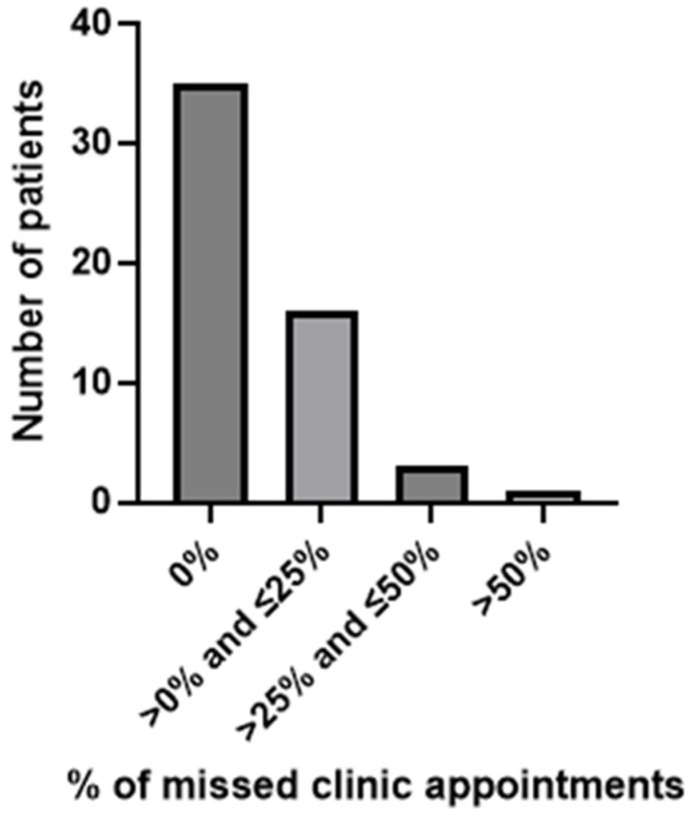
Frequency of missed clinic appointments.

Patient engagement was measured indirectly by assessing (1) the percentage of scheduled clinic appointments missed by the patient and (2) the proportion of patients who activated their online patient portals during the navigation program. Figure 5 illustrates appointment attendance. Many of the evaluable patients (35/55; 63.6%) did not miss any scheduled clinic appointment (Figure 5). Meanwhile, 16 patients missed up to 25%, 3 missed 25–50%, and only 1 missed >50% (55.6%; 5/9 appointments) of all scheduled clinic appointments. Notably, the four patients with the highest percentages of missed clinic appointments (55.6%, followed by 50%, 42.1%, and 33.3%) all had a cancer diagnosis at enrollment. Moreover, all four reported transportation concerns and had received a transportation gift card to help resolve this barrier. Specifically, the highest rate of appointments missed (55.6%) was characterized by a late stage of lung cancer, >2 barriers identified, and higher neighborhood disadvantage. Patients with transportation and Internet access concerns were significantly more likely to miss one or more scheduled clinic appointments than those who did not have these barriers (*p* = 0.023 and 0.035, respectively; Table S5). Other sociodemographic variables (gender, age, ethnicity, distance travelled), clinical variables (diagnosis, lung cancer stage), and barriers (psychosocial concerns, financial concerns, current smoking habit) were not significantly associated with missed appointment rates (*p* > 0.05; Tables S5 and S6). While the variable race was explored, the QI study design, scope of the project, small number of patients included, and the complex nature of race as a sociopolitical construct, as well as the lack of consideration for socioeconomic status, racism, or social determinants of health, threaten a valid statistical interpretation. Historically, race has been pathologized, medicalized, and misrepresented in the literature, which drives inappropriate bias if responsible evaluation is not considered and planned [46,47,48,49]. Because of the limited sample size to control for confounding, further regression analyses are beyond the scope of this QI project. Thus, we have chosen not to highlight race alone in the context of our QI project to avoid perpetuation of race-based inequalities in the absence of contextual drivers discussed above.

Access to COH’s online patient portal is offered to all new patients at COH-AV upon registration. However, 26 of the evaluable patients in this study had not signed up for the patient portal. Notably, after meeting with the navigator, 18 of these patients signed up for the online patient portal, giving a conversion rate of 69.2%.

## 4. Discussion

Findings from our QI project suggest that the development and execution of a barrier-focused telephone-based lay lung navigator program is feasible in a low-resource setting. Key findings identified during program evaluation were (1) anticipated practical barriers such as transportation and Internet challenges were among the top three needs addressed; (2) frequency and intensity of navigator encounters were most notable for patients with cancer; (3) higher complexity of needs required longer navigation times; (4) positive patient engagement was observed in appointment attendance and online patient portal activation during the course of the navigator program; and (5) transportation concerns and Internet access concerns were significantly associated with appointment attendance.

More than 76% of our evaluable patients were 65 years and older, consistent with the statistics for the overall US population, wherein 83% of patients with lung cancer are diagnosed at ≥65 years of age [50]. Participants were largely White and non-Hispanic; there were no persons who identified as Native American/Alaskan, Asian, or Asian Pacific Islanders. However, racial and ethnic demographics within our study population did not aptly mirror the composition of the general AV population, which is composed of nearly 45% Hispanic, 14% Black (non-Hispanic), and 4% Asian individuals [32]. This discrepancy may have arisen due to multiple reasons, including the observation that non-Hispanic White individuals have the highest rates of lung cancer screening eligibility as defined by various guidelines [51]. Many patients in the program lived in disadvantaged neighborhoods with limited to no access to thoracic surgeons compared to less disadvantaged neighborhoods. This was likely to be concerning for our patients who had stage I or II disease where lung surgery is likely to be part of the treatment plan. Moreover, barriers like a lack of transportation and Internet access are particularly challenging for persons living far from cancer care. Notably, 25% of our program participants lived >50 miles from local cancer care services, and more than 90% lived >50 miles from our large volume thoracic surgery center (COH-Duarte). Transportation insecurity remains a significant barrier for many adults with cancer, particularly among older adults or those with limited social support [52,53]. In a study of lay navigation among older cancer patients, patients were more likely to ask for help with questions about insurance/financial needs (79%) and transportation (76%) when these items contributed to distress [54]. Similarly, patients in our program expressed concerns related to transportation and financial security. Thus, telehealth proposes a viable alternative vehicle to access care. Telehealth-based navigation in resource-limited settings, while challenging, has previously been shown to be successful [55]. A study showed that older patients in California are more likely to use telehealth than other age groups, which may be because they are more likely to access the healthcare system for visits [9]. Interestingly, in our program, while Internet-enabled devices were offered, less than 40% of those who were interested in borrowing these devices took advantage of this resource.

Frequency and intensity of encounters were greater among patients with cancer and complex care needs. We made efforts to capture navigation intensity (relative time needed for navigation) as opposed to navigation acuity, which is “a measure of patient distress, medical and psychosocial barriers (i.e., financial toxicity, comorbidities, lack of family support), and the complexity of illness and social determinants that indicate the need for the intensity of subsequent navigator interventions across the care continuum” [56]. In this study, we observed a wide range of navigation intensity among participants, which was closely linked to the number of barriers identified. Similarly, one recent study of lay navigation to facilitate treatment completion among 139 breast and colorectal cancer patients found that 63% of the patients had one or more barriers at baseline (similar to our observation of barriers in 60% of evaluable patients) [45]. Moreover, this study found that navigation intensity increased with the number of barriers, similar to our observations. Furthermore, an umbrella review of systematic reviews found navigation intensity in cancer care to range from 5 min to 3 h per session [57]. Thus, the mean navigation intensity of 23 min noted in our study, and the observation that more barriers need longer navigation times, were both comparable to other navigator programs associating complexity with longer navigation times [58]. Lastly, we observed favorable patient engagement overall in appointment attendance and online patient portal activation during the delivery of the navigator program. Most patients in our program attended their scheduled clinic appointments, and activation of online patient portals may have been favorably induced by navigator engagement with patients. Our findings are similar to other studies that used navigators to boost portal activation [59] and attendance with appointments [57].

We highlight several key lessons learned from our experience in delivering a lay navigator program. Firstly, while we offered services related to transportation and Internet access to several participants with these barriers, we encountered challenges in resource utilization among these participants. During the setup and initial execution of the program, we discovered that rideshare cards, such as those for Uber^®^ and Lyft^®^, were restricted by county boundaries. For example, if a patient received care in Los Angeles County but lived in a neighboring county, they could not redeem the rideshare cards. In response to this issue, during one of our PDSA cycles, we switched to offering Visa^®^ gift cards for gas. However, gas cards required access to a vehicle or social support with transportation, which limited their effectiveness. This may explain why four patients, who had cancer as well as transportation needs, still had the highest rate of missed appointments despite receiving Visa^®^ gift cards through our program. Secondly, we anticipated that the navigator program would be most frequently utilized by patients with lung cancer and those who required thoracic surgery. However, we found that the frequency of patient–navigator encounters, particularly crisis encounters and after-hours encounters, was higher among patients with stage IV disease. In these cases, our navigator provided emotional support and assisted with palliative care needs in collaboration with the healthcare team. Additionally, our navigator, who was a skilled life coach, earned certification as a professional navigator through the American Cancer Society Navigator credentialing program at the conclusion of the study period. Thus, our navigator’s adeptness in navigating patients with advanced cancer needs may be unique. Thirdly, most of the literature regarding patient–navigator utilization focuses on screening, treatment, and surveillance among patients with cancer [57]. However, based on our experience, lay navigators may be particularly useful for older adults with advanced cancer receiving palliative care. Finally, there is growing interest in how geographic data, such as neighborhood disadvantage, may impact cancer health outcomes [60,61]. These “geomarkers” or objective, contextual, or geographic measures that may impact clinical outcomes have been studied in many different populations and communities to further inform decision-making and resource needs [62,63]. Moreover, consideration of “geomarkers” or geographic measures such as community neighborhood assets and lay navigation has been well studied globally in social prescribing models—the concept of connecting patients through use of navigators with non-clinical support and services within their communities, integrating health and social care [64,65]. Most programs successfully adopting this concept have largely been in England and Canada because of government-funded healthcare models, compared to the US, which has a largely diverse, complex healthcare system. However, social prescribing initiatives are increasingly being implemented [64,65]. Nieman et al. describe a QI project in Missouri, USA, using social prescribing with navigation as an intervention. They noted that among 22 participants, 11 (50%) accessed at least one of the resources provided through social navigation, and 9 (21%) participated in an activity offered by the resource accessed [66]. This illustrates a promising integrated approach to maximizing available community assets and navigation for potential barrier intervention. However, in low-resource communities, further exploration would be needed.

Our participants included individuals at various points in the cancer care continuum from pre-cancer diagnosis to active cancer diagnosis and treatment. They also varied by location of residence (rural vs. urban), which greatly affected access to cancer services. The majority of the participants were White and from socioeconomically deprived communities with fewer healthcare resources, such as large-volume thoracic surgical centers. This observation underscores the need for context-informed care beyond race and ethnicity, with attention to low-resource community inhabitants of all backgrounds.

While our insights are useful to guide the next steps (beyond feasibility) of a lay navigator program for low-resource communities, we note several limitations to our study. These include the following: (1) a small sample size and the QI nature of our program, both of which severely restrict conclusions or the generalizability of our results beyond the COH patient population evaluated in the study; (2) the short duration of program delivery, which may have restricted buy-in and integration within the healthcare system; (3) concurrent institutional changes, such as hiring a new patient intake navigator and a clinic site nurse navigator, which may have influenced uptake; (4) the lack of program buy-in from external providers to COH-AV, as evidenced by lack of referrals; (5) offering iPad^®^s instead of laptops/computers to resolve the Internet access barrier, which may have presented a technological challenge for some participants as tablets may be less intuitive to use; (6) the high proportion of patients 65 years of age or older, which could have resulted in decreased utilization of resources and referrals offered by our navigator, especially technology-dependent resources such as iPad^®^s and transportation gift cards; (7) the telephonic nature of the majority of sensitive patient–navigator interactions, which could have introduced vulnerabilities into privacy safeguards or caused hesitation among patients who may have been using a phone shared with another family member; (8) eligible patients were English speaking only in this pilot program, which may have limited the program’s reach to Latinos/Latinas or persons of different race and ethnic backgrounds where English is a second language with lung cancer or suspicious radiographic findings; (9) the quality and intervention fidelity of navigation were not evaluated during our QI project; and (10) the lack of planned program-specific outcomes such as patient satisfaction using validated tools (such as the Patient Satisfaction with Interpersonal Relationship with Navigator scale) [67].

## 5. Conclusions

Our telehealth-based barrier-focused lay lung navigator program delivered in a low-resource community was patient-centered and feasible. While our study does not provide definitive conclusions due to its QI design, it provides insight and guidance for our COH enterprise—with clinical practice networks spanning southern California and the greater Chicago (Illinois), Atlanta (Georgia), and Phoenix (Arizona) areas—to address the needs and optimize healthcare delivery for the urban and rural communities we serve. As expected, patients with a higher number of identified barriers, as well as those with a lung cancer diagnosis at enrollment, had a higher frequency and intensity of encounters with the navigator. Barriers to transportation and Internet access were significantly associated with missed appointment rates in our study population. While addressing practical barriers, such as transportation and Internet access, may be important, we noted that older patients with advanced cancer, particularly those receiving palliative care, may benefit from emotional support and coordination of care. Navigation of these specific populations, particularly in rural areas, warrants further exploration with particular emphasis on the role of social prescribing and navigation. Adopting such models at COH-AV offers a promising strategy to incorporate geographic context into the planning, resource mapping, and assessment of navigation programs in low-resource communities served by COH clinical practice networks.

## Figures and Tables

**Table 1:** Patient demographic and clinical information.

Demographic/Clinical Information	Number of Evaluable Patients (N = 55)
**Gender-N (%)**	
Male	24 (43.6%)
Female	31 (56.4%)
**Age (years)**	
Median	71
**Population Distribution by Age-N (%)**	
<65 years	13 (23.6%)
≥65 years	42 (76.4%)
**Race-N (%)**	
Black	7 (12.7%)
White	46 (83.6%)
Unknown	2 (3.6%)
**Ethnicity-N (%)**	
Hispanic or Latino	≤5 (≤9.1%)
Not Hispanic or Latino	50 (90.9%)
Unknown	≤5 (≤9.1%)
**Smoking Status-N (%)**	
Current	15 (27.3%)
Former	32 (58.2%)
Never	8 (14.5%)
**Diagnosis-N (%)**	
High-risk lung nodule/mass with no cancer diagnosis	17 (30.9%)
Lung cancer	38 (69.1%)
**Lung Cancer Stage-N (%)**	
I	9 (23.7%)
II	7 (18.4%)
III	9 (23.7%)
IV	13 (34.2%)
**Patient Travel Distance (miles)-Median (Interquartile Range)**	
Residence to COH-AV	26 (14–58)
Residence to COH-Duarte	93 (82–122)
**Population Distribution by Travel Distance-N (%)**	
Residence to COH-AV ≤ 50 miles	41 (74.5%)
Residence to COH-AV > 50 miles	14 (25.5%)
Residence to COH-Duarte ≤ 50 miles	≤5 (≤9.1%)
Residence to COH-Duarte > 50 miles	≥50 (≥90.9%)

**Table 2:** Frequency of different types of barriers among enrolled patients.

Patient Category	Total Number of Enrolled Patients N (%) N = 55
**Patients who had ≥1 barrier**	33 (60.0%)
***Barriers for which referrals were offered***	
**Patients with psychosocial concerns**	
Concerned about paying rent/mortgage	≤5 (≤9.1%)
Has childcare responsibilities	≤5 (≤9.1%)
Needs to talk to a mental health professional	9 (16.4%)
Is the primary caretaker of someone disabled or elderly	≤5 (≤9.1%)
Concerned about current living situation	≤5 (≤9.1%)
**Patients with financial concerns about paying for medical care**	13 (23.6%)
**Patients who are current smokers**	15 (27.3%)
**Patients with barriers who were offered ≥1 referral**	31 (56.4%)
***Barriers for which resources were offered***	
**Patients with Internet access concerns** ^#^	13 (23.6%)
**Patients with transportation concerns**	17 (30.9%)
**Patients with barriers who received resources**	
Loaner iPad^®^s	5 (38.5%)
Gas-Gift Cards	12 (70.6%)

^#^ Patients who were interested in borrowing a loaner iPad^®^ for telehealth appointments were deemed as having Internet access concerns.

## Data Availability

The original contributions presented in this study are included in the article. Further inquiries can be directed to the corresponding author.

## Supplementary Materials

The following supporting information can be downloaded at: https://www.mdpi.com/article/10.3390/curroncol32090491/s1, Table S1: Questionnaire used by the navigator to conduct needs assessment; Table S2: Program outputs and process outcomes used to evaluate the navigator program; Table S3: Demographic characteristics of patients recruited through provider referral vs. navigator screening; Table S4: Number of barriers by demographic and clinical variables; Table S5: Appointment adherence by the presence or absence of specific barriers; Table S6: Appointment adherence by demographic and clinical variables.

## Abbreviations

The following abbreviations are used in this manuscript:

ADI	Area Deprivation Index
AV	Antelope Valley
CITI	Collaborative Institutional Training Initiative
COH	City of Hope
HIPAA	Health Insurance Portability and Accountability Act
PDSA	Plan-Do-Study-Act
QI	Quality Improvement
