# Supplementary material for: Insights from a Patient-Centered Lung Cancer Navigation Program in a Low-Resource Community
Source: Curr Oncol. 2025 Sep 1;32(9):491. doi: 10.3390/curroncol32090491 (PMC12468844; doi:10.3390/curroncol32090491)
Supplement: Supplementary file 1 [file curroncol-32-00491-s001.zip › curroncol-3729203-supplementary.pdf]

Supplementary Information

Insights from a Patient-centered Lung Cancer Navigation Program in a Low-resource Community

Tanyanika Phillips, MD, MPH[1,*]; Anjaney Kothari, PhD[2]; Africa Robison[3]; Jeffrey Mark Erfe, MD, MPH[2]; Dan J Raz, MD, MAS[2]

[1]Department of Medical Oncology and Developmental Therapeutics, City of Hope National Medical Center, Duarte, CA 91010, USA

[2]Division of Thoracic Surgery, Department of Surgery, City of Hope National Medical Center, Duarte, CA 91010, USA

[3]Sheri and Les Biller Patient and Family Resource Center, Department of Supportive Care Medicine, City of Hope National Medical Center, Duarte, CA 91010, USA

*Corresponding Author:

Tanyanika Phillips, MD, MPH

Assistant Professor

Department of Medical Oncology and Developmental Therapeutics

City of Hope National Medical Center, 1500 E Duarte Road, Duarte, CA 91010

Phone: +1-626-218-2067; Email: taphillips@coh.org

Table S1. Questionnaire used by the navigator to conduct needs assessment.

**Transportation**	
Do you have any concerns about getting a ride to any of your upcoming medical appointments?	o Yes<br>o No
Transportation notes	
**Internet access**	
Which of the following devices do you have access to at home?	☐ Computer (desktop or laptop)<br>☐ Tablet (such as iPad)<br>☐ Smartphone where you can browse the internet
Do you have access to reliable internet at home?	o Yes<br>o No
Would you be interested in borrowing an internet-enabled laptop computer for telehealth appointments?*	o Yes<br>o No<br>o Not applicable (has internet/device)
[City of Hope patients only] Have you signed up for the City of Hope patient portal?	o Yes<br>o No<br>o Not applicable (non-COH)
Internet access comments	
**Financial concerns**	
Are you concerned about being able to pay for your medical care?	o Yes<br>o No
Would you like to speak to one of our financial counselors?	o Yes<br>o No
Financial concerns comment	
**Psychosocial needs**	
Would you consider where you are currently living to be temporary, such as staying with a friend or relative, a hotel or motel, group home, shelter, or vehicle?	o Yes<br>o No
Comments on current living situation	
Are you worried about how you are going to pay your rent or mortgage?	o Yes<br>o No
Are you worried you may face eviction?	o Yes<br>o No
Are you the primary caretaker of someone who is disabled or elderly?	o Yes<br>o No
Do you have childcare responsibilities that make your medical care challenging?	o Yes<br>o No
Are you experiencing or have you recently experienced physical or emotional abuse from a partner?	o Yes<br>o No
Would you like to talk to a mental health professional for any reason? (such as	o Yes<br>o No

depression, feeling overwhelmed with diagnosis, etc.)	
Psychosocial needs comment	
**Smoking status**	
Does the patient currently smoke? (cigarettes only)	o Yes<br>o No
If the patient is currently smoking cigarettes, is the patient interested in referral to smoking cessation?	o Yes<br>o No<br>o Not applicable
Smoking comments	

**While this question was initially framed to ask about laptop computers, the navigator modified it to tablets/iPad®s prior to program initiation as the budget only allowed for iPad®s.*

Table S2. Program outputs and process outcomes used to evaluate the navigator program.

**Program Outputs**	**How Outputs/Outcomes Were Assessed**
Number of patients identified for and enrolled in the navigation program	Patients referred to the program by providers and those identified by the navigator were noted separately.
Number of navigation encounters	All encounters, including navigator-directed and patient-directed encounters, whether telehealth or in-person, were recorded to determine frequency.
Intensity of navigation encounters	Intensity was measured as the total length of time of all encounters involving a patient; follow-up/referral time was not included.
Number of patients with barriers	Barrier assessment tool (Table S1) was used to determine the number of patients who had one or more barriers.
Number of barriers identified	Barrier assessment tool (Table S1) was used to determine the number of barriers identified for each patient. This could range from 0 to 11.
Number of patient services and referrals offered	Services included transportation gift cards and internet-enabled iPad®s. Referrals included referrals to social workers, financial advisors, and tobacco cessation counselors. The number of services accepted, and the number of referrals offered were recorded. Follow-up on whether a patient actually visited the social worker/financial advisor/tobacco cessation counselor they were referred to was not conducted.
**Process Outcomes**	
Number (and percentage) of appointments missed	The navigator manually reviewed appointment schedules and recorded the

	total number of scheduled clinic appointments as well as the number of appointments missed by the patient. Appointments missed due to provider or clinic-related issues were recorded separately but not included in the analysis.
Number of patients who activated their online patient portals, and conversion rate	The number of patients who had not signed up for the COH patient portal prior to enrollment, but signed up for it upon navigation, was determined. Conversion rate was calculated as the percentage of patients who had not signed up for the portal who signed up for it upon navigation.

Table S3. Demographic characteristics of patients recruited through provider referral vs. navigator screening.

Demographic variable		Referred by providers (N = 30)	Identified by the navigator (N = 38)	p-value[#]
**Gender**	**Male**	9	17	0.161
	**Female**	21	21	
**Age**	**<65**	9	6	0.134
	**≥65**	21	32	
**Race**	**White**	24	34	0.368
	**Black**	≤5[^]	≤5[^]	
	**Unknown**	≤5[^]	≤5[^]	
**Ethnicity**	**Hispanic or Latino**	≤5[^]	≤5[^]	0.859
	**Not Hispanic or Latino**	27	33	
	**Unknown**	≤5[^]	≤5[^]	

[#] *Fisher's exact test used; one-sided p-value calculated for 2x2 contingency tables.*

[^] *Small cell counts (≤5) have been suppressed to maintain k-anonymity.*

Table S4. Number of barriers by demographic and clinical variables.

Demographic/Clinical Variable	Number of Evaluable Patients			p-value#
	No barrier	1-3 barriers	4-7 barriers	
**Gender**				
Male	9	13	≤5^	0.746
Female	13	17	≤5^	
**Age (years)**				
<65 years	5	7	≤5^	>0.999
≥65 years	17	23	≤5^	
**Race**				
Black	≤5^	≤5^	≤5^	0.668
White	20	23	≤5^	
Unknown	≤5^	≤5^	≤5^	
**Ethnicity**				
Hispanic or Latino	≤5^	≤5^	≤5^	0.411
Not Hispanic or Latino	22	25	3	
Unknown	≤5^	≤5^	≤5^	
**Diagnosis**				
High-risk lung nodule/mass with no cancer diagnosis	10	7	≤5^	0.144
Lung cancer	12	23	≤5^	
**Lung Cancer Stage**				
I	≤5^	6	≤5^	0.951
II	≤5^	≤5^	≤5^	
III	≤5^	≤5^	≤5^	
IV	≤5^	8	≤5^	
**Patient Travel Distance to COH-AV (miles)**				
≤ 50 miles	16	23	≤5^	0.894
> 50 miles	6	7	≤5^	
**Patient Travel Distance to COH-Duarte (miles)**				
≤ 50 miles	≤5^	≤5^	≤5^	0.006*
> 50 miles	22	28	≤5^	

#Fisher's exact test used.

**p<0.05 (statistically significant).*

^Small cell counts (≤5) have been suppressed to maintain k-anonymity.

Table S5. Appointment adherence by the presence or absence of specific barriers.

Barrier category	Number of Evaluable Patients		p-value#
	No appointments missed	≥1 appointments missed	
**Psychosocial concerns**			
Concerned about paying rent/mortgage			
Yes	≤5^	≤5^	0.703
No	>30^	>15^	
Has childcare responsibilities			
Yes	≤5^	≤5^	0.364
No	>30^	>15^	
Needs to talk to a mental health professional			
Yes	≤5^	≤5^	0.423
No	30	16	
Is the primary caretaker of someone disabled or elderly			
Yes	≤5^	≤5^	0.364
No	35	19	
Concerned about current living situation			
Yes	≤5^	≤5^	0.131
No	34	17	
**Financial concerns about paying for medical care**			
Yes	8	5	0.553
No	27	15	
**Current smokers**			
Yes	9	6	0.483
No	26	14	
**Internet access concerns**			
Yes	5	8	0.035*
No	30	12	
**Transportation concerns**			
Yes	7	10	0.023*
No	28	10	

#Fisher's exact test used; one-sided p-value calculated for 2x2 contingency tables.

**p<0.05 (statistically significant).*

^Small cell counts (≤5) have been suppressed to maintain k-anonymity.

Table S6. Appointment adherence by demographic and clinical variables.

Demographic/Clinical Variable	Number of Evaluable Patients		p-value[#]
	No appointments missed	≥1 appointments missed	
**Gender**			
Male	17	7	0.245
Female	18	13	
**Age (years)**			
<65 years	8	5	0.553
≥65 years	27	15	
**Race[&]**			
Black	0	7	0.0002*
White	34	12	
Unknown	≤5^	≤5^	
**Ethnicity**			
Hispanic or Latino	≤5^	≤5^	0.484
Not Hispanic or Latino	33	17	
Unknown	≤5^	≤5^	
**Diagnosis**			
High-risk lung nodule/mass with no cancer diagnosis	>10^	≤5^	0.154
Lung cancer	22	16	
**Lung Cancer Stage**			
I	>5^	≤5^	0.090
II	≤5^	≤5^	
III	≤5^	≤5^	
IV	≤5^	>5^	
**Patient Travel Distance to COH-AV (miles)**			
≤ 50 miles	24	17	0.153
> 50 miles	>10^	≤5^	
**Patient Travel Distance to COH-Duarte (miles)**			
≤ 50 miles	≤5^	≤5^	0.463
> 50 miles	>50^	>15^	

[#] *Fisher's exact test used; one-sided p-value calculated for 2x2 contingency tables.*

**p<0.05 (statistically significant).*

^ *Small cell counts (≤5) have been suppressed to maintain k-anonymity.*

[&] *While the variable race was explored, the Quality Improvement study design, the limited scope of the project, the small number of patients included, the complex nature of race as a sociopolitical construct, and the lack of consideration for socioeconomic status or social determinants of health does not allow for a valid statistical interpretation.*
